# Supplementary material for: First Report of Enterocytozoon hepatopenaei Infection in Pacific Whiteleg Shrimp (Litopenaeus vannamei) Cultured in Korea
Source: Animals (Basel). 2021 Nov 4;11(11):3150. doi: 10.3390/ani11113150 (PMC8614486; doi:10.3390/ani11113150)
Supplement: Supplementary file 1 [file animals-11-03150-s001.zip › animals-1366114-supplementary.pdf]

Supplementary Table S1. The spore wall protein (SWP) gene similarity between this study with others in the top ten blast matches

Accession NO.	Scientific Name	Description	Similarity (%)
MG015710	Enterocytozoon hepatopenaei	Enterocytozoon hepatopenaei spore wall protein 1 (SWP1) mRNA, complete cds	100
KX258197	Enterocytozoon hepatopenaei	Enterocytozoon hepatopenaei spore wall protein 1 (SWP) gene, partial cds	100
MW000460	Enterocytozoon hepatopenaei	Enterocytozoon hepatopenaei isolate C spore wall protein gene, partial cds	100
MW000459	Enterocytozoon hepatopenaei	Enterocytozoon hepatopenaei isolate B spore wall protein gene, partial cds	100
MW000458	Enterocytozoon hepatopenaei	Enterocytozoon hepatopenaei isolate A spore wall protein gene, partial cds	100
MN308364	Enterocytozoon hepatopenaei	Enterocytozoon hepatopenaei spore wall protein gene, partial cds	100
KY674357	Enterocytozoon hepatopenaei	Enterocytozoon hepatopenaei spore wall protein 1 gene, partial cds	100
MH365434	Enterocytozoon hepatopenaei	Enterocytozoon hepatopenaei spore wall protein 1 gene, partial cds	100
KY593132	Enterocytozoon hepatopenaei	Enterocytozoon hepatopenaei spore wall protein gene, partial cds	100
KY593133	Enterocytozoon hepatopenaei	Enterocytozoon hepatopenaei spore wall protein gene, partial cds	100
